# Future Intentions Regarding Quitting and Reducing Cigarette Use in a Representative Sample of Canadian Daily Smokers: Implications for Public Health Initiatives

**DOI:** 10.3390/ijerph7072896

**Published:** 2010-07-19

**Authors:** John A. Cunningham, Peter L. Selby

**Affiliations:** Centre for Addiction and Mental Health, 33 Russell St., Toronto, Ontario, M5S 2S1, Canada; E-Mail: Peter_Selby@camh.net

**Keywords:** tobacco cessation, smoking reduction, epidemiology, population survey

## Abstract

Pre-cessation reduction is associated with quitting smoking. However, many smokers reduce the amount consumed but may not quit altogether. Using a representative sample of adult current daily smokers, this project explored future intentions of smokers regarding cigarette consumption. This information is important because it can provide a framework within which to plan tobacco cessation initiatives. A random digit dialing telephone survey was conducted of 889 Canadian current daily smokers, 18 years and older. The response rate was 65% (of households with a smoker in residence, 65% agreed to participate). Analyses focused on the 825 respondents who smoked at least 10 cigarettes per day at some point in their lives. As part of this survey, respondents were asked their future plans about their smoking (maintain, increase, reduce, quit). Of these 825 respondents, the majority of respondents had plans to change their cigarette use, with 55% planning to quit, 18.8% to reduce and 22.5% to maintain the amount they smoked (3.4% did not know and 2 respondents planned to increase). Most smokers who planned to reduce their smoking saw it as a step towards quitting smoking completely. These results present a picture of smokers, the majority of whom appear to be in some form of transition. Many smokers planned to reduce, of which the overwhelming majority saw their reduction as a step towards quitting. Opportunities exist to capitalize on these intentions to change in efforts to promote tobacco cessation.

## 1. Introduction

What pathways do smokers take in changing their cigarette use? While the majority of research has focused on quit attempts and successes, several studies have reported general population data on smokers reducing the amount they smoke as well as on their attempts to quit. Using separate longitudinal general population samples, West *et al.* [1] and Meyer *et al.* [2] found considerable numbers of smokers who were able to reduce the amount they smoked and to maintain this reduction over time. Similarly, in a follow-up of smokers who failed to quit in the Lung Health Study, Hughes and colleagues [3] found many participants were able to reduce their cigarette consumption and further, to maintain these reductions over a four year period. A cross-sectional US sample [4] found that more than half of smokers had tried to reduce smoking in the past and one-quarter planned to reduce in the next year. In addition, using newspaper recruited samples, Hughes and colleagues [5,6] found that more than three-quarters of smokers were as interested (or more interested) in gradual cessation as compared to abrupt cessation.

While there has been no evidence to-date that smokers’ attention on reducing the amount they smoked precluded attempts to quit smoking, there is still concern that promoting reduction as an option for smokers might delay or eliminate their intentions to quit smoking [7,8]. In addition, with smokers’ ability to compensate in the way they smoke to offset the reduction of nicotine that can result from reducing the number of cigarettes smoked [9], there is also justifiable concern with recommending a behaviour change that has little or no health benefit. However, a review by Hughes [8] outlined the evidence collected to-date on these issues and concluded that attention to reduction as a smoking goal has value because there is evidence that reductions in smoking are positively associated with quit attempts in the future. Further, a recent Cochrane Systematic Review [10] concluded that there was support for the use of reduction goals as part of tobacco cessation interventions.

The other important reason for studying reduction in the general population of smokers is the fact that smokers engage in this behaviour. Without a clear picture of what smokers are planning to do, how can we adequately plan a population or clinical approach to promoting tobacco cessation? The present study adds to the growing literature on smoking reductions by exploring intentions about future reduction and quit attempts. We also investigated what smokers meant when they said that they had a reduction goal. Mainly, what was the goal, when were they planning to reduce, and was this reduction the endpoint or did they see their reduction as a pathway towards tobacco cessation?

## 2. Methods

A random digit dialing telephone survey was conducted of 889 Canadian current daily smokers, 18 years and older (survey conducted June–September, 2006). Interviews were conducted by the Institute for Social Research, York University using Computer Assisted Telephone Interviewing technology. Interviews were offered in English or French. Households with landline telephones were contacted and asked if there was a daily smoker who lived in residence. Up to 12 call backs were made at different times of the day and week to each telephone number until contact was made. The person contacted was then asked if there were any daily smokers living in the house and, if so, whether one of them was willing to participate in a telephone survey. If more than one adult smoker lived in the household the smoker with the most recent birthday was selected. The estimated response rate was 65% (889/1,372 households estimated to contain at least one daily smoker).

Respondents were first asked about their current smoking status (number of cigarettes smoked per day; time between waking and first cigarette), age at their first cigarette, age when they started smoking daily, and what type of cigarettes they currently smoked (*i.e.*, regular, light, mild). Respondents were also asked about their future intentions regarding smoking. They were told that, “people have plans about what they are going to do about their smoking. What are your plans about smoking? Are you: going to maintain the current amount you smoke, increase the amount you smoke, reduce the amount you smoke, or are you planning to quit?” Respondents who said they were planning to quit were then asked if they planned to reduce first or if they planned to quit all at once. Respondents who said they were planning to reduce were asked if they planned to reduce as a step towards quitting.

Finally, a series of demographic characteristics were asked. The survey took an average (SD) of 26.8 (7.4) minutes to complete. Proportions, means and statistical values are based on the weighted sample to make them representative of the general population of Canadian smokers. Sample sizes are presented as unweighted values. The study was approved by the standing research ethics board of the Centre for Addiction and Mental Health.

## 3. Results

### 3.1. Characteristics of the Sample

Of the 889 respondents interviewed, we will focus on the 825 who had smoked at least 10 cigarettes a day at some point in their lives. The decision was made to restrict the analyses to this subsample in order to ensure that all respondents had been established smokers at some point in their lives (of the 64 excluded, 60 never smoked 10 or more cigarettes per day and 4 currently smoked less than 10 cigarette per day but could not recall if they ever smoked more). Respondents had a mean (SD; range) age of 43.9 years (14.1; 18–81), roughly half (47.7%) were male and 57.5% were married or in common law relationships. The majority of respondents reported a family income of CAN$30,000 or more (70.8%), 46.7% had at least some postsecondary education and 65.8% were full or part time employed. Respondents smoked an average (SB) of 18.0 (8.8) cigarettes per day.

### 3.2. Future Intentions Regarding Change

**Figure 1.**
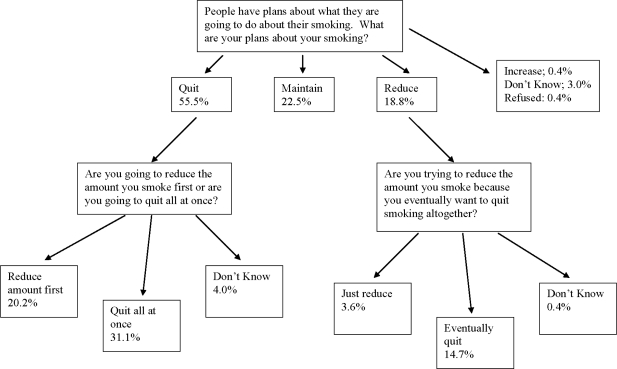
Future Intentions Regarding Smoking.

Most respondents had plans to make changes to their smoking in the future. When asked what their plans were about their smoking, 55% said they planned to quit, 18.8% said they planned to reduce and 22.5% said they were going to maintain (3.4% did not know or refused; 2 respondents said they were going to increase). Interestingly, when asked if they were going to quit all at once or to reduce first, roughly one-third of the respondents who said they were going to quit said they planned to reduce first. Similarly, of respondents who said they planned to reduce as an initial response, almost four-fifths said they were reducing as a means to eventually quit. Figure 1 below summarizes the future intentions of this group of smokers (note: all proportions are based on the entire sample, N = 825).

Table 1 displays the demographic and smoking characteristics of three groups of respondents—those who said they plan to maintain (22.5% of sample), those who plan to reduce (18.8% of sample), and those who plan to quit (55% of sample). The three groups were relatively similar on demographic characteristics with the only significant differences being that respondents who intended to quit were younger, F(2,773) = 9.1, *p* < 0.001, and less likely to be married, χ^2^(2) = 9.9, *p* < 0.001, as compared to those who intended to reduce. In contrast, there were some noticeable differences in smoking characteristics between the three groups. First, those who planned to quit were smoking fewer cigarettes as compared to those who planned to maintain, F(2,784) = 6.8, *p* = 0.001. Second, those who planned to reduce started smoking at an older age than those who planned to maintain, F(2,778) = 6.0, *p* < 0.01. Third, those who planned to quit had been smokers for fewer years than those who planned to maintain or reduce their smoking, F(2,752) = 9.0, *p* < 0.001. Taken together, these results tend to paint a picture of smokers who plan to quit as having smoked for a shorter period of their lives as compared to those who planned to maintain or reduce. However, these differences are small so their clinical significance is questionable. Recall also that most smokers who said they planned to reduce also said they were planning to reduce as a step towards quitting. However, the response of those smokers who say they are going to reduce as their first step might be indicative of their longer smoking careers and marginally heavier smoking as compared to those that first say they were planning to quit.

Finally, respondents who planned to reduce the amount they smoked were asked how many cigarettes per day they would be smoking when they met their goal of reduction. While respondents who planned to quit reported their reduction goal as being less than those who planned to reduce (Mean [SD] number of cigarettes will smoke once reach goal = 5.6 [4.8] *vs.* 7.5 [6.6], t-test, 235.7 df = 2.7, *p* < 0.01), the actual amount each group planned to reduce was not significantly different (Mean [SD] number of cigarettes plan to reduce = 11.0 [6.9] *vs.* 10.8 [7.0], *p* > 0.05).

## 4. Discussion

This survey caught a ‘snapshot’ of smokers in transition. Almost three-quarters of smokers were planning some type of change in the future. Smoking reductions were a major component of change plans. These reductions were often explicitly seen as a pathway to quitting. One-third of respondents who said they planned to quit agreed that they intended to reduce first when they were probed about their intentions regarding change. Almost 80% of respondents who said they planned to reduce stated that they were reducing as part of their plan to quit smoking when they were similarly probed regarding the details of their intentions to change. Only one-tenth of respondents who said they planned to reduce had no current intentions to quit smoking.

Current evidence and guidelines suggest abrupt discontinuation of smoking is necessary for smokers to be successful quitters regardless of whether they quit using pharmacotherapy or behavioural methods alone [11]. Although reduction in the number of cigarettes smoked is not associated with any measurable health benefit [12], behaviourally reductions might be associated with eventual cessation [13] mediated by an increase in self-efficacy.

There were several limitations to this study. First, a screening question at the beginning of the survey asked for daily smokers and it is unknown to what extent this question led to limitations in the generalizability of the data (*i.e.*, how many potential smoking respondents were lost because the person answering the telephone said there were no daily smokers in the house when in fact there were?). A second limitation to this study is that the results are based on a cross-sectional sample of smokers reporting on their own intentions. Longitudinal research looking at actual changes in smoking can provide much stronger evidence regarding the role of reductions. In fact, prior research in this area has employed longitudinal data sets [1–3], lending confidence to the validity of the current findings. This study attempted to add to this extant literature by probing in greater depth what smokers’ intentions regarding reductions and quitting mean.

Despite these limitations, what is clear from this study is that plans regarding smoking reductions are common. What can’t be stated from this study is whether reductions should be recommended in a public health tobacco control policy. Should such a policy encourage people to reduce smoking because it is recognized that most reductions are intended as a step to quitting? But then what to do about those who reduce but don’t go on to quit? And would suggesting that some people reduce their smoking lead to a ‘redirection’ of a proportion of people who would have tried to quit towards reducing instead? However, smoking reduction goals should certainly be recognized and their implications for a public health policy towards tobacco cessation further studied because such reductions are a common phenomenon and are seen by smokers as a pathway towards tobacco cessation. The challenge remains to orient the trajectory to a full cessation from smoking so that smokers do not accrue health consequences while stopping and starting to smoke.

## Figures and Tables

**Table 1.** Demographic and smoking characteristics among those who plan to maintain, reduce or quit smoking.

**Variable**	**Maintain (N = 175)**	**Reduce (N = 163)**	**Quit (N = 456)**	***p***
Mean (SD) Age	44.4 (14.1)	47.7 (14.4)	42.2 (13.6)	0.001
% Male	49.5	40.3	49.3	>0.05
% Married/Common law	60.8	67.3	53.4	0.01
% Family income CAN$30,000 or more	74.5	72.9	69.5	>0.05
% Some postsecondary education	48.4	45.1	46.0	>0.05
% Full/part time employed	71.5	59.9	66.3	>0.05
Mean (SD) Cigarettes/day	19.8 (9.4)	18.6 (9.1)	17.0 (8.5)	0.001
% Smoked first cigarette <5 min waking	33.9	28.2	24.8	>0.05
Mean (SD) Age smoked first cigarette	13.8 (3.9)	15.4 (3.9)	14.6 (4.2)	0.01
Mean (SD) Years a daily smoker	27.8 (13.7)	29.5 (14.1)	24.6 (13.1)	0.001
% Smoking Light/Mild cigarettes	50.5	61.0	62.7	0.02

## References

[1] West R McEwen A Bolling K Owen L Smoking cessation and smoking patterns in the general population: a 1-year follow-up Addiction 2001 96 891 902 11399220 10.1046/j.1360-0443.2001.96689110.x

[2] Meyer C Rumpf HJ Schumann A Hapke U John U Intentionally reduced smoking among untreated general population smokers: prevalence, stability, prediction of smoking behaviour change and differences between subjects choosing either reduction or abstinence Addiction 2003 98 1101 1110 12873244 10.1046/j.1360-0443.2003.00475.x

[3] Hughes JR Lindgren P Connett J Nides M Smoking reduction in the Lung Health Study Nicotine Tob. Res 2004 6 275 280 15203800 10.1080/14622200410001676297

[4] Shiffman S Hughes J Ferguson SG Pillitteri JL Gitchell JG Burton SL Smokers’ interest in using nicotine replacement to aid smoking reduction Nicotine Tob. Res 2007 9 1177 1182 17978992 10.1080/14622200701648441

[5] Hughes JR Callas PW Peters EN Interest in gradual cessation Nicotine Tob. Res 2007 9 671 675 17558824 10.1080/14622200701365293

[6] Hughes JR Smokers who choose to quit gradually *versus* abruptly Addiction 2007 102 1326 1327 17624983 10.1111/j.1360-0443.2007.01948.x

[7] Hatsukami DK Henningfield JE Kotlyar M Harm reduction approaches to reducing tobacco-related mortality Ann. Rev. Pub. Health 2004 25 377 395 15015926 10.1146/annurev.publhealth.25.102802.124406

[8] Hughes JR Carpenter MJ Does smoking reduction increase future cessation and decrease disease risk? A qualitative review Nicotine Tob. Res 2006 8 739 749 17132521 10.1080/14622200600789726

[9] Hatsukami DK Le CT Zhang Y Joseph AM Mooney ME Carmella SG Hecht SS Toxicant exposure in cigarette reducers *versus* light smokers Cancer Epidemiol. Biomarkers Prev 2006 15 2355 2358 17164356 10.1158/1055-9965.EPI-06-0240 PMC6512339

[10] Lindson N Aveyard P Hughes JR Reduction *versus* abrupt cessation in smokers who want to quit Cochrane Database Syst Rev 2010 3 CD008033 20238361 10.1002/14651858.CD008033.pub2

[11] Fiore M Bailey WC Cohen SJ Clinical Practice Guideline Panel Treating Tobacco Use and Dependence: Clinical Practice Guideline US Department of Health and Human Services Rockville, MD, USA 2000

[12] Tverdal A Bjartveit K Health consequences of reduced daily cigarette consumption Tob. Control 2006 15 472 480 17130377 10.1136/tc.2006.016246 PMC2563668

[13] Hyland A Levy DT Rezaishiraz H Hughes JR Bauer JE Giovino GA Cummings KM Reduction in amount smoked predicts future cessation Psychol. Addict. Behav 2005 19 221 225 16011395 10.1037/0893-164X.19.2.221

